# Disrupted connectivity in the olfactory bulb-entorhinal cortex-dorsal hippocampus circuit is associated with recognition memory deficit in Alzheimer’s disease model

**DOI:** 10.1038/s41598-022-08528-y

**Published:** 2022-03-15

**Authors:** Morteza Salimi, Farhad Tabasi, Maryam Abdolsamadi, Samaneh Dehghan, Kolsoum Dehdar, Milad Nazari, Mohammad Javan, Javad Mirnajafi-Zadeh, Mohammad Reza Raoufy

**Affiliations:** 1 Department of Physiology, Faculty of Medical Sciences, Tarbiat Modares University, Tehran, 1411713116 Iran; 2 Faculty of Medical Sciences, Institute for Brain Sciences and Cognition, Tarbiat Modares University, Tehran, Iran; 3 Department of Mathematics, Faculty of Science, Islamic Azad University, North Tehran Branch, Tehran, Iran; 4 Stem Cell and Regenerative Medicine Research Center, Iran University of Medical Sciences, Tehran, Iran; 5 Eye Research Center, The Five Senses Institute, Rassoul Akram Hospital, Iran University of Medical Sciences, Tehran, Iran; 6 Department of Molecular Biology and Genetics, Aarhus University, Aarhus, Denmark; 7 DANDRITE, The Danish Research Institute of Translational Neuroscience, Aarhus University, Aarhus, Denmark

**Keywords:** Alzheimer's disease, Learning and memory, Neural circuits, Neuroscience, Olfactory bulb

## Abstract

Neural synchrony in brain circuits is the mainstay of cognition, including memory processes. Alzheimer's disease (AD) is a progressive neurodegenerative disorder that disrupts neural synchrony in specific circuits, associated with memory dysfunction before a substantial neural loss. Recognition memory impairment is a prominent cognitive symptom in the early stages of AD. The entorhinal–hippocampal circuit is critically engaged in recognition memory and is known as one of the earliest circuits involved due to AD pathology. Notably, the olfactory bulb is closely connected with the entorhinal–hippocampal circuit and is suggested as one of the earliest regions affected by AD. Therefore, we recorded simultaneous local field potential from the olfactory bulb (OB), entorhinal cortex (EC), and dorsal hippocampus (dHPC) to explore the functional connectivity in the OB-EC-dHPC circuit during novel object recognition (NOR) task performance in a rat model of AD. Animals that received amyloid-beta (Aβ) showed a significant impairment in task performance and a marked reduction in OB survived cells. We revealed that Aβ reduced coherence and synchrony in the OB-EC-dHPC circuit at theta and gamma bands during NOR performance. Importantly, our results exhibit that disrupted functional connectivity in the OB-EC-dHPC circuit was correlated with impaired recognition memory induced by Aβ. These findings can elucidate dynamic changes in neural activities underlying AD, helping to find novel diagnostic and therapeutic targets.

## Introduction

Alzheimer's disease (AD) is a progressive neurodegenerative disease and one of the most common causes of dementia with devastating morbidity and mortality^1^. AD is characterized by escalating cognitive decline, commonly anterograde amnestic cognitive impairments. Patients present with varying manifestations depending on the disease stage and primarily affected structures^2^. However, AD patients frequently present with deficits in recognition memory^3^, which notably can occur in prodromal stages as an early cognitive symptom^4,5^. Therefore, altered recognition memory is potentially associated with changes in regions' activities earlier than significant changes in volume or architecture, which could serve as biomarkers.

Several hypotheses have been introduced to explain the etiopathogenesis and clinical course of AD. The amyloid hypothesis is a widely accepted explanation based on extracellular aggregation and deposition of amyloid-beta peptides (Aβ) as a causal factor in AD^6^. The Aβ interferes with neuronal homeostasis, causes synaptic dysfunction and dysregulated neural excitability^7^, and triggers other pathological events^8,9^, leading to cognitive impairments. Compelling evidence demonstrated that cognitive impairment in AD might not be related to neuronal degeneration alone but also occur due to deficits in synchronized neural activities^10,11^. However, little is known about neural connectivity in circuits related to recognition memory.

Organized communications and information transfer during memory processes in both local circuits and distant networks in the brain are allocated by synchronized rhythmic activities^10^. AD is associated with significantly disrupted connectivity in such neuronal communications^12^, which can be present from early preclinical stages before progression to overt Alzheimer's dementia^13^. Notably, AD tends to involve specific neural assemblies and networks that are more vulnerable to Aβ aggregates^14^. Two structures that are proposed affected in AD early stages are the entorhinal cortex (EC)^15^ and hippocampus (HPC)^16^, both pivotal for proper memory functions, notably, object recognition^17^. Functional and structural changes in EC and HPC can be detected years before significant clinical symptoms, and therefore, suggested as potential biomarkers for AD diagnosis^18^.

Recent evidence indicates that olfactory network dysfunction may precede hippocampal formation damages^19–21^. Olfactory dysfunction is shown to be present as a disturbed sense of smell, proposed as an early sign of neurodegenerative diseases, including AD^21^. Functional changes in the olfactory system, particularly the olfactory bulb (OB), are shown in AD patients, with or even without smell problems^21^. Thus, OB can be a key candidate for investigating early functional changes in the brain induced by AD pathology.

Notably, the OB rhythmic oscillations, which are phase-locked to respiration, are global, propagate throughout the brain, coupled to fast cortical oscillations (e.g., gamma) and most importantly, synchronize distant regions' activities^22–25^. Therefore, OB could be considered a pivotal regulator of neural activity in the brain during cognitive performances^26,27^. On the other hand, the OB is closely connected with the EC-HPC circuit, which has synchronized oscillatory activity during spatial recognition^22,28^. Hence, exploring the OB network in recognition memory can provide useful information about dynamic functional connectivity alterations in AD.

**Figure 1:**
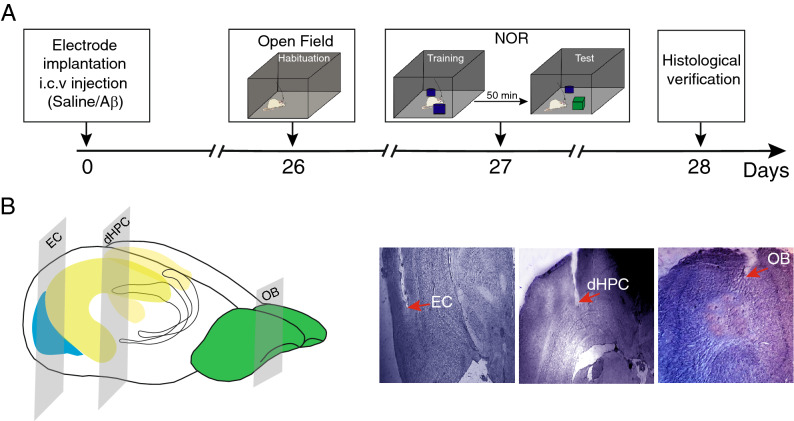
Experimental protocol and histological verification. (**A**) Schematic representation of the study timeline. Animals received bilateral i.c.v injection of saline or Aβ 4 μg in 2 μl. After recovery from surgery and model induction, animals were placed in the open field for habituation at day 26 and NOR task, with one day apart. (**B**) 3D view of electrode implantation sites on the brain and histological confirmation. i.c.v, intracerebroventricular;* Aβ* amyloid-beta,* NOR* novel object recognition,* OB* olfactory bulb,* dHPC* dorsal hippocampus,* EC* entorhinal cortex.

The novel object recognition (NOR) task is widely accepted to evaluate memory deficits in AD animal models^29^. This task is based on the natural propensity of the animal to explore a new object, more than a familiar one, and does not concern spatial reference memory^30^. Given close structural and functional connections between OB and hippocampal formation, we hypothesized that the Aβ could disrupt OB-EC-HPC circuit connectivity in the AD rat model, resulting in recognition memory impairment. Therefore, to explore the circuit's connectivity in NOR performance, we simultaneously recorded local field potentials (LFPs) from OB, EC, and dorsal HPC (dHPC) during NOR task performance in an animal model of AD (Fig. 1 illustrates study protocol). This study can provide a better understanding of task-related dynamic circuit connectivity in AD, particularly in those regions that are more vulnerable to Aβ aggregates.

## Results

### Histopathological findings

**Figure 2:**
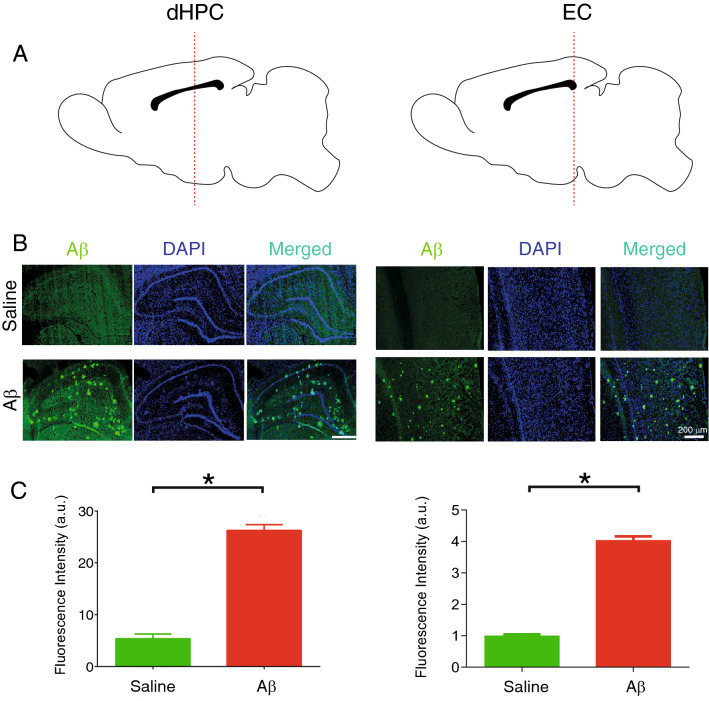
Aβ plaques accumulation in regions of interest of AD model animals. (**A**) Schematic sections for immunostaining in the dHPC and EC (**B**) Immunostaining sections from animals received i.c.v saline and Aβ. Immunostaining of anti-Aβ_1-42_ indicated a high level of plaque accumulation in Aβ animals compared to the saline group. **(C)** Fluorescence intensity (a.u.) is significantly higher in Aβ animals compared to the saline group. White bar scale indicating 200 µm.* Aβ* amyloid-beta,* AD* Alzheimer’s disease, DAPI, 4′,6-diamidino-2-phenylindole;* dHPC* dorsal hippocampus; a.u., arbitrary units.

**Figure 3:**
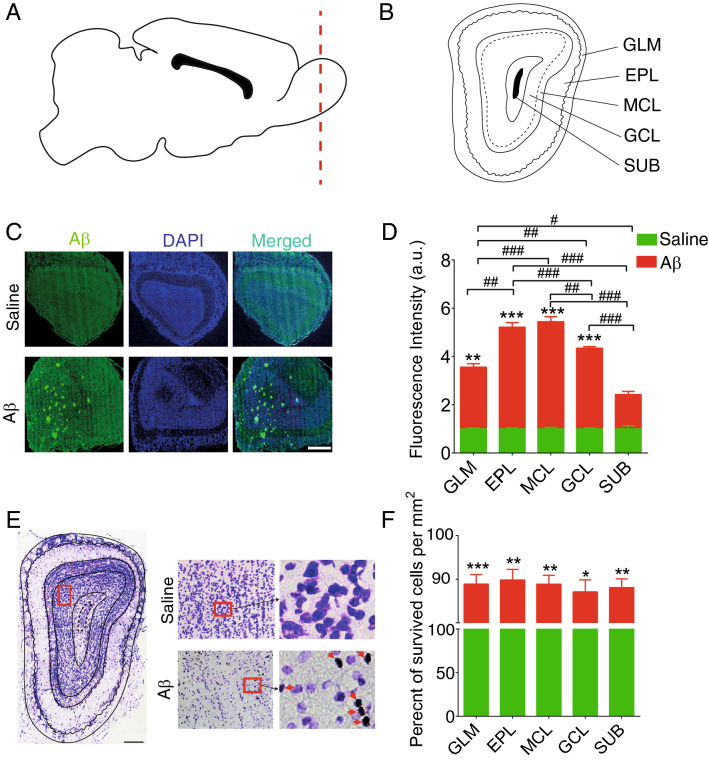
Survived cells in the OB. (**A** and **B**) Schematic representation of OB subregions. (**C**) a sample of immunostaining sections from OB of animals that received i.c.v. saline and Aβ. (**D**) Immunostaining of anti-Aβ_1-42_ indicated a high level of plaque accumulation in Aβ animals compared to the saline group. (**E**) Representative of OB section stained by Nissl method to evaluate the survived cells in OB subregions. (**F**) Aβ animals demonstrate significantly lower survival cells in this region compared to the saline group. Red arrows show dense bodies of dead cells.* Aβ* amyloid-beta,* OB* olfactory bulb,* GCL* granule cell layer,* GLM* glomerular layer,* EPL* external plexiform layer,* MCL* mitral cell layer,* SUB* subependymal layer, *p < 0.05, **p < 0.01, ***p < 0.001 compared to saline. #*p* < 0.05, ##*p* < 0.01, ###*p* < 0.001 comparision among OB subregions in Aβ group.

Immunostaining of Aβ_1–42_ of EC and dHPC sections demonstrated a high level of Aβ plaques in Aβ animals compared to the saline group (Fig. 2). This result verifies our model induction. Moreover, we evaluated the pathology of Aβ as well as the number of survived cells in the OB subregions in both groups. As shown in Fig. 3, generally animals that received Aβ showed a significant increase in plaque accumulation. Our findings showed that Aβ pathology affected granule cell layer (GCL), glomerular layer (GLM), external plexiform layer (EPL), and mitral cell layer (MCL) containing glomerular, mitral, and tufted cells rather than the rest of the subregions. Similarly, the number of survived cells in the Aβ group is significantly lower than in the saline group, illustrating that the Aβ caused a degenerative condition in the OB. Focusing on subregions illustrates no significant changes across different subregions of OB in the Aβ group. Altogether, these results demonstrate that Aβ_1-42_ infusion results in Aβ plaque formation and neural loss, hallmarks of AD pathology in our region of interests (i.e., OB, EC, and dHPC), and confirm model induction.

### Recognition memory performance

The ability to recognize a previously exposed entity can be used to determine memory alterations. Due to innate exploratory behavior, animals naturally tend to spend more time exploring a novel object than a familiar object, meaning animals remember a previously explored object^31^. The novel object recognition (NOR) evaluates an animal's behavior in exposure to a novel and a familiar object by assessing the discrimination index (DI)^30^. NOR can be used for evaluating different types of memory, including recognition memory.

**Figure 4:**
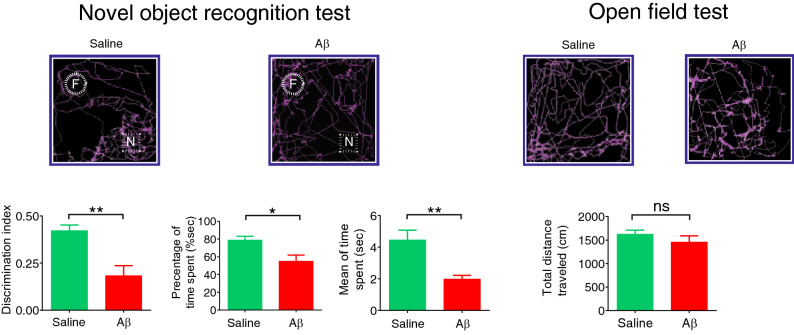
Aβ animals showed recognition memory impairment. (Left) Top panels indicate tracking points during the NOR task. The bottom panels denote that the preference ratio for the novel object was significantly attenuated in Aβ animals, measured by discrimination index, percentage by time spent, and mean of time spent. (Right) Aβ and saline animals showed no significant differences in the total traveled distance as an indicator of locomotor activity. Bar graphs represent mean ± SEM. The comparison was conducted by t-test, n = 6 per group. *p < 0.05, **p < 0.01, ***p < 0.001, ****p < 0.0001 Aβ compared to saline. *Aβ* amyloid-beta,* F* familiar object,* N* novel object,* NOR* novel object recognition.

Moreover, when the novel object was tested 50 min after training, we found that Aβ animals spent significantly less time exploring the novel object (mean ± SEM of time: 1.98 ± 0.23 s vs. 4.45 ± 0.61 s, *p* = 0.003) and more time exploring the familiar object than rats that received saline (average percentage of time spent for the novel object in Aβ and control animals was 54.94% and 78.76%, respectively. *p* = 0.015; mean ± SEM of DI in Aβ and control animals was 0.18 ± 0.05 and 0.42 ± 0.03, respectively, *p* = 0.001; Fig. 4). However, no significant changes were found for traveled distance in the open field box (mean ± SEM of total traveled distance in Aβ and control animals was 1459 ± 131.3 cm and 1627 ± 85.06 cm, respectively, *p* = 0.31), indicating locomotor activity was intact and not different among groups. These results showed that recognition memory is impaired in animals that received Aβ compared to the control group.

### Aβ dropped coherence in OB-EC-dHPC circuit during NOR task performance

**Figure 5:**
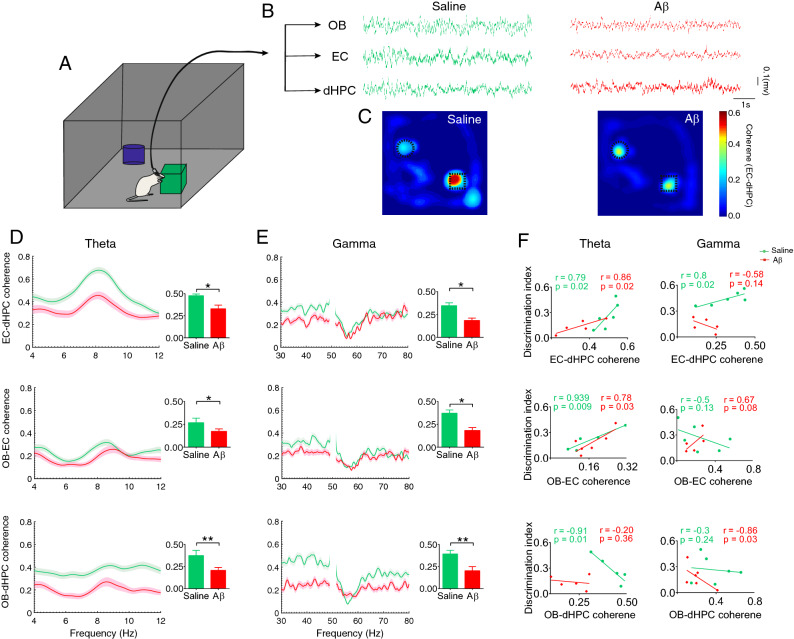
Aβ reduced coherence in the OB-EC-dHPC circuit during recognition memory performance. (**A**) A schematic illustration of the rat while exploring the novel object. (**B**) A representative sample of signals when animals performed NOR tasks. (**C**) A sample of EC-dHPC coherence on maze during the task. LFPs are binned into the positional frame, and the mean pixel coherence is color-coded to illustrate coherence on the maze. Darker color indicates enhanced coherence beside the novel object. (**D** and **E**) Coherence spectral in OB-EC-dHPC circuit at theta and gamma frequency bands, respectively. Compared to the saline group, Aβ animals showed a significant reduction in the coherence at both theta and gamma. (**F**) Correlation between recognition memory performance and OB-EC-dHPC coherence. NOR task performance (measure with discrimination index) was correlated with coherence in the OB-EC-dHPC circuit. At theta band, the correlation between NOR performance and coherence in EC-dHPC and OB-EC was positive for both groups and negative in OB-dHPC for the saline group. At the gamma band, the correlation between NOR performance and coherence in EC-dHPC was positive in the saline group. Also, in OB-EC and OB-dHPC circuits, this correlation was positive and negative for Aβ animals, respectively. Lines display the mean of coherence, and the shaded area represents SEM. Bar graphs are generated by mean values of coherence. Comparisons were made by t-test and Pearson correlation coefficients. For Pearson correlation, data were assessed regarding outliers. n = 6 per group. *p < 0.05, **p < 0.01. *Aβ* amyloid-beta,* OB* olfactory bulb,* EC* entorhinal cortex,* dHPC* dorsal hippocampus,* LFP* local field potential,* NOR* novel object recognition.

The EC-dHPC is a critical circuit for memory performance, particularly object recognition memory^17,32^. Accordingly, to explore whether Aβ affects the interaction between EC-dHPC during recognition memory, we examined the coherence of the circuit during NOR task performance (Fig. 5A–C). Our analysis showed animals that received Aβ had significantly lower coherence values in EC-dHPC at theta and gamma bands (*p* = 0.004 and *p* = 0.0014, respectively; Fig. 5D,E, upper sections) while exploring the novel object. On the other hand, OB sends axonal projection to the hippocampal formation areas, particularly to the EC^33^. Therefore, we evaluated the functional connectivity between OB and EC-dHPC during recognition memory performance. In Aβ animals, the coherence of OB with EC and dHPC was significantly reduced at both theta and gamma frequency bands (OB-EC: *p* = 0.04 and *p* = 0.001, respectively and OB-dHPC: *p* = 0.004 and *p* = 0.001, respectively; Fig. 5D,E middle and bottom sections). These results indicate that Aβ can disrupt the functional connectivity in the OB-EC-dHPC circuit, notably in theta and gamma bands.

We then assessed the correlation between recognition memory performance and coherence in the OB-EC-dHPC circuit to find a possible relationship between LFPs and the behavior (Fig. 5F). Accordingly, DI (as a measure of NOR performance accuracy) was positively correlated with EC-dHPC coherence for the saline group at theta and gamma (r = 0.79, *p* = 0.02, and r = 0.8, *p* = 0.02, respectively), and for Aβ animals at theta (r = 0.86, *p* = 0.02). In the OB-EC circuit, this correlation was significantly positive for the saline group at theta (r = 0.939, *p* = 0.009) and also for Aβ animals at both theta and gamma (r = 0.79, *p* = 0.03, and r = 0.67, *p* = 0.08, respectively). Finally, in the OB-dHPC circuit, the results showed a negative correlation for the saline group at theta (r = − 0.91, *p* = 0.01) and for Aβ animals in gamma (r = − 0.86, *p* = 0.03).

### Synchrony in OB-EC-dHPC circuit during recognition memory performance

**Figure 6:**
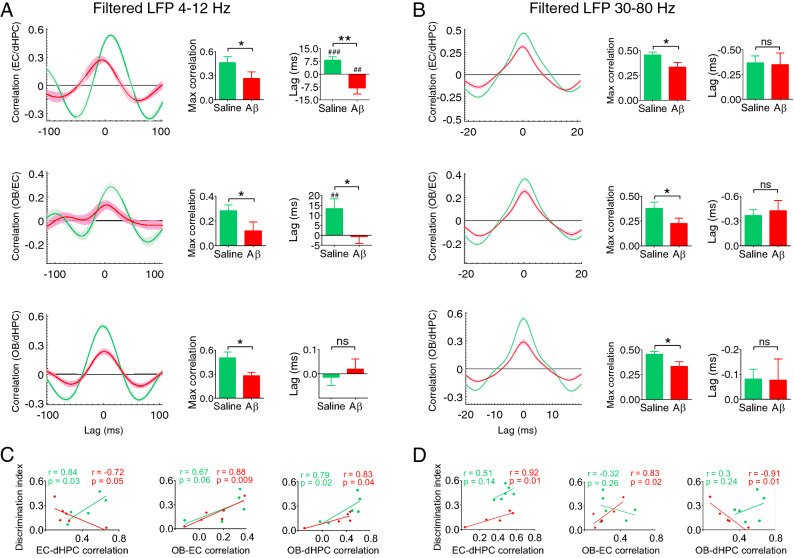
Aβ disrupts synchrony in the OB-EC-dHPC circuit during recognition memory performance. (**A** and **B**) Mean correlation in time lag at theta and gamma bands, respectively. The network correlation in Aβ animals decreased at both theta and gamma ranges compared to the saline group. A reduction was found at the theta band for information influx lag from OB to EC and EC to dHPC in Aβ animals. Moreover, Aβ animals showed an inverted direction of information influx from OB to EC and EC to dHPC at theta band. (**C** and **D**) Correlation between task performance and synchrony at theta and gamma bands, respectively. A correlation was found between NOR performance and synchrony in the OB-EC-dHPC circuit at theta and gamma bands. At theta band, NOR performance was positively correlated with OB-dHPC and OB-EC synchrony for both groups. Also, in the EC-dHPC circuit, this correlation was negative and positive for Aβ animals and the saline group, respectively. At the gamma band, the correlation was negative between NOR performance and circuits for Aβ animals. Lines display the mean of correlation coefficient across the lag, and the shaded area represents SEM. Bar graphs are generated by an average of maximum coefficient and lag values for cross-correlation. Comparisons were conducted by t-test and Pearson correlation coefficients. n = 6 per group. For Pearson correlation, data were assessed regarding outliers. *p < 0.05, **p < 0.01; ns, not significant. ##*p* < 0.01, ###*p* < 0.001 according to one-sample t-test comparison with the constant value of zero. *Aβ* amyloid-beta,* OB* olfactory bulb,* EC* entorhinal cortex,* dHPC* dorsal hippocampus,* NOR* novel object recognition.

To address the effect of Aβ on signal synchrony, we explored the time-lagged cross-correlation in the OB-EC-dHPC circuit. During NOR performance, Aβ animals exhibited a significant drop of cross-correlation at both theta and gamma bands in EC-dHPC (*p* = 0.048 and *p* = 0.025, respectively), OB-EC (*p* = 0.043 and *p* = 0.047, respectively), and OB-dHPC (*p* = 0.012 and *p* = 0.006, respectively) circuits (Fig. 6A,B). We then computed the lag of information influx in this circuit. Similarly, our analyses indicated a significant reduction in the lag of information influx from OB to EC (*p* = 0.014) and EC to dHPC (*p* < 0.001) in Aβ animals compared to the control group. These results show that the Aβ impaired the amount and the direction of information influx.

Moreover, we found that NOR performance in Aβ animals was correlated with cross-correlation (referred to as correlation in Fig. 6C,D) in the OB-EC-dHPC circuit at theta and gamma frequency bands: In the EC-dHPC circuit, for Aβ animals, DI was negatively and positively correlated with cross-correlation at theta (r = − 0.72, *p* = 0.05) and gamma (r = 0.92, *p* = 0.01), respectively; for the saline group, this correlation was significantly positive at theta range (r = 0.84, *p* = 0.03). Regarding the OB-EC circuit, the correlation between NOR performance and cross-correlation was positive for both Aβ and saline groups at theta (r = 0.88, *p* = 0.009, and r = 0.67, *p* = 0.06, respectively) and for Aβ animals at gamma (r = 0.83, *p* = 0.02). In the OB-dHPC circuit, DI was correlated with cross-correlation positively for both Aβ and saline groups at theta and gamma (r = 0.83, *p* = 0.04, and r = 0.79, *p* = 0.02, respectively), and negatively for the Aβ group at gamma (r = − 0.91, *p* = 0.01). These observations suggested that Aβ strongly disrupts synchrony and direction of information influx within OB-EC-dHPC regions, which is correlated with NOR performance.

## Discussion

The current study explored the OB-EC-dHPC circuit connectivity during the NOR task in the rat model of AD. The EC-dHPC is a critical circuit for object recognition^17,32^. Moreover, the OB is closely connected with hippocampal formation, particularly EC, a hub for the hippocampus^28,33,34^. Initially, we found that coherence and synchrony of the EC-dHPC circuit are decreased in Aβ animals compared to the control group at theta and gamma bands during NOR task performance. Further, we assessed the OB connectivity with the EC-dHPC circuit. Aβ, demonstrate impaired object recognition performance. Further, we found that Aβ reduced coherence and synchrony of OB-EC and OB-dHPC at theta and gamma frequencies during task performance. Notably, we revealed that disrupted functional connectivity in the OB-EC-dHPC circuit is correlated with recognition memory impairment induced by Aβ. Our results provide direct evidence regarding Aβ-induced synchrony disruption in the OB-EC-dHPC circuit during the NOR task, which is associated with impaired NOR.

Synchronized neural activities in brain circuits are essential for organized cognitive functions, which are affected in patients with AD^10,13^. These pathological events can be observed from the early stages of the disease, known as mild cognitive impairment (MCI)^13,35^. AD patients exhibit altered organized communication in both local and long-range circuits associated with cognitive deficits^11^. Several studies in these patients demonstrate changes in functional connectivity of oscillatory activities at resting-state and also during cognitive performances^36,37^. For instance, AD patients showed decreased global synchrony in alpha, beta, and gamma frequency bands during resting-state^38^. Also, in task-related neural synchrony, AD patients showed lower synchronization in alpha, and beta ranges activity during information maintenance of working memory^39^. This evidence demonstrated a disconnected state in the brain's networks occurring in AD^11^.

AD pathology has a propensity to alter the function and structure of specific brain regions^14^. For instance, change in hippocampal synchrony occurs in the prodromal stages of AD, which is proposed as an early biomarker for the disease^13^. Moreover, hypotrophic changes in EC and HPC can be detected in MCI patients, who progressed to Alzheimer's dementia^40^. In this line, the EC is known as one the earliest regions affected by AD^41,42^, and its dysfunction can be responsible for initial memory symptoms^43^. The EC is implicated in converging spatial and sensory information^44^, and alterations in its function due to AD may explain why these patients are showing deficits in recognition memory at preclinical stages^45^. Consistent with previous observations, the present study demonstrated that coherent oscillatory activity in EC-dHPC was correlated with NOR task performance. Also, we found that Aβ animals with impaired NOR performance showed remarkably lower coherence in this circuit at both theta and gamma ranges. This indicates coherent activity at low (theta) and high (gamma) frequencies between EC-dHPC is required for object recognition memory. In Aβ animals, we found disrupted oscillatory coupling at theta and gamma ranges between EC and dHPC associated with impaired object recognition.

Recent evidence suggests that pathologic and degenerative changes in OB may occur even before EC damage^19,20^. This is based on observations that hyposmia and anosmia occur in preclinical AD, before significant memory symptoms^46,47^, and are associated with olfactory network impairments^48^. Consequently, OB involvement can induce pathological changes in connected structures, including EC and HPC^48^.

Intact OB oscillations, notably in the gamma band, are required for odor-related processing and memory and learning processes^49^. The OB is closely connected with regions responsible for spatial coding and memory processes, such as EC via direct reciprocal connections^33^ and HPC via EC via the perforant pathway^50^. Therefore, changes in OB activities can influence EC and HPC functions. Our results illustrated that oscillatory coupling in OB-EC and OB-dHPC at theta and gamma range is required for NOR. Also, task performance was correlated with the circuit's synchronized activity at the theta and gamma range. Accordingly, Aβ animals in our study demonstrated disrupted synchrony in theta and gamma range in OB connection with EC-dHPC circuit during task performance. One paradoxical results of the current study were that in some frequency ranges a negative correlation between connectivity parameters and object recognition performance was found. Our findings were consistent with the former observation claiming that connectivity between brain circuits involving the hippocampus, rather than being directly associated with memory performance, is reflective of a ‘compensatory response for preserving optimal performance’^51^. Thus, animals with less memory function had employed more values of connectivity at least in some frequency ranges to improve the impaired function. We assumed that this effect may not only be restricted to the memory impairment model but also animals with intact memory performance could recruit the brain oscillation for cognitive performance. However further investigations are required to address how brain function gained neural oscillations.

It has been previously shown that soluble Aβ can impair OB activities^52^. The Aβ is a potential synaptic regulator, which acts as positive and negative regulators in presynaptic and postsynaptic activities, respectively^53^. Aβ accumulation can change the proportion of abnormally hypo- and hyperactive neurons in cortical circuits^12^, inducing synaptic decline^52^. On the other hand, it has been postulated that synaptic activity can modulate the Aβ levels, particularly in the interstitial fluid^54^. For instance, gamma oscillations are suggested to counteract amyloid load and AD-associated pathology by inducing neuronal and glial responses^55^. Moreover, interactions between subregions of OB cells play an important role in generating oscillations at a wide range of frequencies particularly theta and gamma bands^56,57^. For example, it has been shown that inhibitory activity in glomerular cells entrained slow oscillations which were temporally coupled with gamma range during odor sniffing^58^. On the other hand, principal cells within the GLM receive glutaminergic input from olfactory neurons. Besides, granule cells in the GCL exert lateral inhibition across larger distances neurons. Frequent sniffing induces slow inhibition in mitral and tufted cells^59–62^ in the EPL and MCL, resulting in faster, gamma range activities in the OB^58^ which can propagate to deep brain structures such as the ventral hippocampus and entorhinal cortex. Our investigation regarding Aβ pathology and the number of alive cells indicated that GLM, EPL and MCL layers containing glomerular, mitral, and tufted cells had dominantly affected by Aβ deposition. Taken together, the relationship between Aβ and OB dysfunction is reciprocal and not one-way: Aβ can impair OB activities, and this impairment can result in Aβ overproduction and accumulation^63^. Several mechanisms can potentially justify our observations regarding impaired neural synchrony induced by Aβ in the OB-EC-dHPC at low- and high-frequency ranges during the NOR task:

*Firstly*, OB rhythmic activity dynamically modulates other brain areas, notably the EC-dHPC, during a cognitive performance^28^. Thus, disrupted coupling due to Aβ in this circuit, probably via its direct effect on OB synapses and regardless of neural loss, may be responsible for impaired recognition memory. Reciprocally, EC damages induced by Aβ can disrupt the activity of OB^21^, which potentially exaggerates connectivity disruption in the circuit.

*Secondly*, OB has been known as a critical center for adult neurogenesis and acts as a hub in differentiating neural precursors for other brain regions^64^. Previously demonstrated that olfactory bulbectomy causes dendritic remodeling and altered synaptic plasticity in EC and HPC, resulting in decreased adult neurogenesis and spatial memory deficits^65^. The olfactory bulbectomy significantly reduced dendritic arborization in EC and reduced neural excitability^66^. Hence, disordered OB architecture and function can induce neuronal degeneration and hypotrophy in adjacent areas, including EC^66^ and HPC^65^. This observation corroborates our assumption that OB rhythmic activity is required for EC-dHPC function and plasticity. The OB-EC functional connectivity can potentially be responsible for reinforcing synaptic plasticity in the circuit, which can be disrupted by Aβ deposition.

*Thirdly*, specific structures in olfactory regions may act as a hub for propagating the proteinopathy in the neurodegenerative diseases to other areas, such as EC and HPC^67^. Aβ could behave prion-like and spread to adjacent areas via neural pathways^68^. Therefore, Aβ accumulation can spread to EC and then HPC, inducing pathological changes in these structures and functions.

It is noteworthy to mention that none of these mechanisms act spontaneously, and a combination is probably responsible for the symptomatology of AD. More studies are required to confirm these mechanisms and their potential application in predicting or even preventing AD complications.

In conclusion, we showed that Aβ disrupts neural synchrony leading to cognitive dysfunction. In particular, this study provided direct evidence regarding disrupted oscillatory connectivity between OB and EC-dHPC circuit at theta and gamma bands in association with impaired NOR performance. Recognition memory deficit related to altered OB-EC-dHPC connectivity can bring attention to the dynamic role of OB in integrating brain activities. We performed this experiment after the fourth week of model induction, considered an early stage of AD. Thus, observed changes in the dynamic activity of OB may be assumed as an early biomarker for AD, although more chronological studies are needed.

Although we showed a marked reduction in OB's cellular population, these results cannot differentiate between functional and non-functional neurons nor concern the alterations in synaptic activities. Further studies are needed to address that AD pathology-induced neural dysfunction in OB can present before irreversible structural damages and overt dementia. Thus, targeting these structures for early diagnosis and therapeutic interventions can prevent further damage and reduce neural injuries associated with cognitive problems. Our findings open a path for more selective targeting of structures, such as OB in AD, given its causal role in organization brain communication.

## Materials and methods

### Animals

The experiments were performed on adult pathogen-free male Wistar rats weighing 210-230 g obtained from Tarbiat Modares University (Tehran, Iran) and housed in 21 ± 2 °C, 12-h light–dark cycle, with free access to food and water. All experiments and procedures were done following the Tarbiat Modares University guidelines for animal care and approved by the Ethics Committee of the Faculty of Medical Sciences, Tarbiat Modares University (IR.MODARES.REC.1398.037). All animal procedures were in accordance with ARRIVE guidelines.

### Surgery and electrode implantation

Rats were anesthetized using intraperitoneal injections of ketamine (100 mg/kg) and xylazine (10 mg/kg) at day 0 (see Fig. 1A for timeline). We placed the anesthetized animals on a stereotaxic apparatus, and a longitudinal incision was made, then the skull was exposed by pulling the skin back. After drilling the skull on intended positions, stainless-steel recording electrodes (127 µm in diameter, A.M. System Inc., USA) were implanted unilaterally according to the stereotaxic coordinates of OB (AP: 8.5 mm, L: − 1 mm, DV: − 1.5 mm), the lateral portion of EC (AP: − 7.04 mm, L: − 5.5 mm, DV: − 6.5 mm) and dHPC (AP: − 3.6 mm, L: − 2.2 mm, DV: − 2.7 mm)^69^. We then implanted a stainless-steel screw at the right side of the parietal bone as a reference point. Finally, acrylic dental adhesive was poured around the electrodes and screws.

To confirm electrodes position, brains were carefully removed and fixed with 4% paraformaldehyde for 48 h. After preparing a 20 μm coronal section using a cryo micro-slicer, we visually compared them with the matching slices of the rat brain atlas^69^ (Fig. 1B). Animals with misplaced electrodes and animals that did not perform the tasks were excluded from the study. Data presented here are taken from twelve rats.

### AD model induction

Aβ aggregation and plaque formation in the brain is one of the hallmarks and key components of AD pathology, which could be responsible for other pathologies^6,70^, including tau aggregation^8,9,71^. Aβ can accumulate intra- and extracellularly, causing devastating impairment in synaptic and neuronal functions, respectively. More importantly, Aβ induces neural apoptosis, resulting in neural degeneration^72,73^. Aβ_1-42_ is a critical agent frequently found in AD patients' brains^70^ that can induce neuronal apoptosis and AD-associated pathology^72,74^.

AD model was induced by Aβ_1–42_ (Cat No. A9810, Sigma-Aldrich, USA) dissolved in normal saline at the concentration of 4 μg/μl. The solution was kept at room temperature for 3 days before administration^75^. Aβ_1–42_ or saline was injected in a volume of 2 μl over 5 min via a microsyringe pump (Stoelting, Lane Dale, IL, USA) connected to the 25-gauge stainless steel needle bilaterally into the lateral cerebral ventricles according to stereotaxic coordination (AP: 0.8 mm, L: ± 1.6 mm, DV: − 3 mm).

### Behavioral assessment

In the assessment locomotor activity and habituation phase of the NOR test, animals were placed individually in the center of the open field box to explore the area freely. To evaluate locomotor activity, animals were individually placed into the open field maze and allowed to explore freely for 10 min. Total traveled was calculated using the graphical interface of MATLAB^76^.

The NOR test is a reliable, sensitive, easy-to-perform behavioral method to evaluate spatial recognition memory^77^ in the AD animal model. This test does not concern the spatial reference memory and avoids inducing excessive stress^29^, negatively affecting memory and learning. Although it is a one-trial test, it consists of two phases: after habituation with the environment, in the first phase, rats first were placed in an open field box (50 cm high, 75 × 75 cm) center containing a couple of the same objects in the opposite corners 10 cm away from walls. Two types of objects, box-shaped (Fig. 1A right) and cylindrical shaped with 10 (diameter) × 20 (height) cm were used in this study (Fig. 1A left). Rats were exploring the objects for 4 min. In the second phase, after a 50-min delay, rats underwent a 4-min test identical to the previous, except one of the training phase objects was replaced with a novel object. Animals with intact memory spend more time beside the novel object than a previously-encountered object^77^. In this regard, we calculated the discrimination index (DI) as follows:

$$\frac{{\left( {{\text{novel object exploration time }} - {\text{ familiar object exploration time}}} \right){ }}}{{\text{total exploration time}}}$$

We also counted the percent and absolute time that animals explored the novel object. To calculate the object exploration time and number, we considered the while that nose-object distance was continuously less than 2.5 cm.

Four visual cues were placed 50 cm away from the mazes. The behavioral performances were recorded using a ceiling-mounted camera above the platform.

### Histopathological assessment

#### Immunofluorescence

Rats were transcardially perfused with cold phosphate-buffered saline (PBS, 10 mM, pH = 7.4), followed by 4% paraformaldehyde in PBS under urethane (1.2 g/Kg) anesthesia. Brains were post-fixed in 4% paraformaldehyde for four hours at 4 °C. Cryoprotection was done with 30% sucrose solution in PBS. Coronal sections through the OB, EC, and dHPC were prepared using a freezing microtome (8 µm thick).

For immunofluorescent labeling, the sections were washed with PBS, permeabilized with Triton- × 100 0.2% for 20 min, and blocked in PBS, 0.2% Triton-X100, 10% normal serum during one hour at RT, followed by overnight incubation with primary antibody for anti-Aβ antibody (A8717, 1:700) at 4 °C. After successive washing with PBS, slides were incubated with proper fluorescent-labeled secondary antibodies (goat anti-rabbit, Alexa Fluor® 488; 1:2000, ab150077) for one hour at room temperature. Nuclei were counterstained with 4',6-diamidino-2- phenylindole (DAPI; Invitrogen Corp., USA), mounted, and images were taken using Olympus fluorescence microscopy and DP72 camera (BX51 TRF, USA).

Four sections of each animal were analyzed using ImageJ software (NIH, USA) for either plaque number or fluorescence intensity quantification. Aβ fluorescence intensity was assessed by measuring the mean gray value of OB, EC, and dHPC areas. Each value was normalized to the mean of tissue background intensity and presented as the percent of the saline group.

#### Nissl staining

Nissl staining was used to evaluate the number of survived cells in both groups. The procedure was performed according to manufacture protocol. Briefly, the sections were rehydrated by graded series alcohols (96%, 80%, and 70%) and stained with 0.1% Cresyl Fast Violet (Merck, Germany) at room temperature for 5 min. Afterward, the sections were washed and then dehydrated by graded series alcohols (70%, 80%, 96%, and 100%) cleared in xylene, cover-slipped using Entellan (Merck, chemical, Germany), and photographed. Images were captured from non-overlapping, consecutive microscopic fields using light Olympus BX-51 microscope and DP72 camera at 400 × magnification. A grid (50 μm × 50 μm) was randomly assigned to the images, and seven squares were counted for survived cells. The number of survived cells is expressed as the number of cells/mm^2^.

### Signal processing

LFPs were simultaneously obtained from OB, EC, and dHPC via a fixed miniature buffer head stage with high-input impedance (BIODAC-A, TRITA Health Technology Co., Tehran, Iran). The signals were amplified using 1000 amplification gain, low-pass filtered < 250 Hz, and digitized at 1 kHz through a recording system (BIODAC-ESR18622, TRITA Health Technology Co., Tehran, Iran)^78,79^. EEG lab toolbox was applied for pre-processing the signals, including noise rejection and baseline correction^80^.

To find whether functional coupling in EC-dHPC circuit during recognition memory performance, we generated an inter-regional coherence map on a NOR maze when rats spontaneously explored the objects. LFP was binned into a positional frame, and the mean pixel coherence was color-coded. Accordingly, we selected 2 s before and 1 s after exploring the novel object. Finally, we averaged the coupling parameters per animal.

To measure the coherence, we computed magnitude-squared coherence using the *mscohere* function of MATLAB. Synchrony in the OB-EC-dHPC network was calculated using cross-correlation analysis, defined as the *xcorr* function in MATLAB software (the "coeff" option was selected for normalizing values). Max values of correlation in each trial were considered as synchrony parameters. All values of maximum cross-correlation were averaged per animal.

### Statistical analysis

GraphPad Prism (version 6.0) was applied to analyze the data and create graphs statistically. The normality distribution of the data was examined via the Kolmogorov–Smirnov test. As all data showed normal distribution, a t-test comparison was made. One sample t-test was applied to compare the mean values with the constant value of 0 or 100. The Pearson's correlation coefficient was applied to assess the correlation between behavioral results and connectivity measures. All data evaluated for the existence of outliers for Pearson's correlation and removed if a significant outlier exists. The *p*-value less than 0.05 was considered statistically significant.

## Data Availability

The datasets generated during and analyzed during the current study are available from the corresponding author on reasonable request.
